# Demographic time bomb in COPD (1990–2050): A joinpoint-APC-BAPC modeling study on diverging risk factor attributions across age and SDI spectrums

**DOI:** 10.1097/MD.0000000000050162

**Published:** 2026-08-14

**Authors:** Dong Liu, Luna Zhao, Lin Chen, Guizhen Lv, Yihao Zhang, Hongding Zhao, Junhao Ma, Hanwen Zhang, Junwei Wang, Kexin Cui, Jingkun Li, Yimiao Qiu, Yi Sun, Lang Wang, Xinxin Zhang, Ye Lui, Fangyi Zhao, Wanting Wang, Luyi Fang, Yuye He, Man Luo, Yue Zhou, Hongzheng Liu, Yun Jia, Sijie Huang, Jinyang Li, Kaiqi Zhang, Rui Zhao, Chao Wu

**Affiliations:** a Department of Pulmonary and Critical Care Medicine, The First Affiliated Hospital of Shihezi University, Shihezi, China; b School of Clinical Medicine, Shihezi University, Shihezi, China; c School of Public Health, Hangzhou Medical College, Hangzhou, China; d School of Basic Medical Sciences, Xi’an Jiaotong University, Xi’an, China; e Department of Pulmonary and Critical Care Medicine, Xinjiang Uygur Autonomous Region People’s Hospital, Urumqi, China; f School of Public Health, Xinjiang Medical University, Urumqi, China.

**Keywords:** aging, COPD, decomposition analysis, environmental pollution, GBD

## Abstract

Chronic obstructive pulmonary disease (COPD) is a leading global cause of morbidity and mortality, characterized by irreversible airflow limitation and progressive symptoms. Despite declining age-standardized rates (ASRs), absolute burdens are rising due to population aging and growth, particularly in low- and middle-Sociodemographic Index (SDI) regions. This study provides the first comprehensive analysis of COPD epidemiology from 1990 to 2021 using Global Burden of Disease 2021 data, integrating multimodel projections to 2050 and risk factor attribution across SDI quintiles. The study extracted COPD incidence, prevalence, mortality, and disability-adjusted life years (DALYs) from the Global Burden of Disease 2021 study (204 countries, 1990–2021). Trends were analyzed via Joinpoint regression, average annual percentage change, age-period-cohort models, and decomposition analysis (population growth/aging/epidemiological shifts). Risk factor burdens (smoking, ambient PM_2_._5_, and household air pollution) were quantified using population attributable fractions. Bayesian age-period-cohort models projected the age-standardized incidence rate, age-standardized mortality rate, and age-standardized DALY rate to 2050. Globally, the age-standardized incidence rate, age-standardized mortality rate, and DALY rate declined (average annual percentage change: −0.05%, −1.49%, and −1.48%, respectively), while absolute cases, deaths, and DALYs increased (213.4 million cases and 1.69 million deaths in 2021). Low-/middle-SDI regions exhibited 2- to 3-fold higher ASRs than high-SDI areas. An inverted V-shaped relationship emerged between SDI and COPD burden, peaking at an SDI of 0.4 to 0.6. Smoking (38%–55% of DALYs), ambient PM_2_._5_ (15%–25%), and household air pollution (0%–55%) dominated risk factors, with marked SDI-based heterogeneity. Projections indicate stable/declining ASRs globally to 2050, but rising absolute burdens in low-SDI nations. COPD remains a critical public health challenge, with shifting burdens from mortality to high prevalence. Targeted interventions in low-SDI regions – clean energy adoption, community spirometry screening, and culturally tailored tobacco control – are imperative to meet the United Nations 2030 noncommunicable disease mortality targets.

## 1. Introduction

Chronic obstructive pulmonary disease (COPD) represents a widespread respiratory disorder marked by persistent and progressive airflow limitations that are not reversible.^[1]^ Commonly, it is exhibited through a lasting cough, production of sputum, and various other respiratory issues, significantly threatening human health and adversely affecting the quality of life (QoL) of those impacted.^[2]^ The symptoms associated with COPD typically consist of long-term and worsening shortness of breath, continual cough, sputum generation, feelings of chest pressure, and exhaustion.^[3]^ In numerous cases, a chronic cough, whether or not accompanied by sputum, may occur intermittently and can precede the onset of airflow restrictions by a number of years.^[4]^

According to the Global Initiative for Chronic Obstructive Lung Disease (GOLD) 2025 guidelines, COPD is diagnosed when the post-bronchodilator forced expiratory volume in 1 second/forced vital capacity ratio falls below 0.70 on a spirometry test.^[5]^ The degree of airflow obstruction is classified into GOLD stages 1 through 4, based on ppFEV_1_ values of ≥80%, 50% to 79%, 30% to 49%, and <30%, respectively, employing the reference equations of the Global Lung Initiative (R Spiro package).^[6]^

Effective COPD management should follow a model that includes diagnosis, initial therapy, evaluation, adjustments, and a spiral-forward treatment approach to meet the goals of stable-phase management.^[7]^ The GOLD 2025 report highlights the importance of recognizing and mitigating risk factors, including considerations related to climate change and COPD.^[5]^ Both extreme temperatures – high and low – are associated with increased mortality rates, particularly the effects of prolonged high temperatures on mortality. Despite the significant morbidity and mortality linked to COPD, the treatment options currently available remain notably few.

COPD affects approximately 384 million people around the world, and estimates indicate that more than half of these individuals may be living with the condition without being diagnosed.^[7]^

COPD ranks as the third leading cause of mortality globally, marked by significant morbidity and mortality rates, which pose a considerable health burden internationally.^[8]^ As such, it is imperative for healthcare providers to consider the possibility of COPD in any patient who presents with chronic symptoms such as dyspnea and cough, especially in those with a history of risk factor exposure, most notably smoking. The diagnosis of COPD should not only be suspected based on clinical signs but must also be confirmed through functional assessments such as spirometry.^[9]^ Moreover, COPD is frequently associated with various chronic comorbidities, including cardiovascular diseases and metabolic disorders, which can adversely affect the overall health outcomes and survival rates of those diagnosed with COPD. The prevalence of this disease notably increases with age, peaking among individuals who are over 60 years old. It is also important to acknowledge that prevalence figures for COPD differ significantly across various countries, which could be attributed to divergent diagnostic criteria employed in different healthcare settings. According to the GOLD definition, the global prevalence of COPD was estimated at 10.3% among individuals aged between 30 and 79 years in 2019.^[10]^ In terms of management, pulmonary rehabilitation has become an established evidence-based therapeutic approach for patients with symptomatic COPD, particularly during stable phases of the disease and following episodes of acute exacerbation. It is recognized as a standard of care to enhance the health and functional capacity of individuals suffering from COPD. There is substantial evidence supporting its effectiveness in alleviating symptoms, improving walking capacity, and enhancing the QoL of these patients. However, it is worth noting that while pulmonary rehabilitation shows positive outcomes in numerous areas, its influence on physical activity levels among COPD patients appears to be somewhat limited.^[11]^

Currently, there is a significant lack of contemporary research focusing on the global dynamic analysis and monitoring of COPD. To address this gap, we undertook a comprehensive update of the data concerning the incidence and mortality rates associated with COPD by examining overall trends from 1990 to 2021, employing the Global Burden of Disease (GBD) framework as our analytical foundation. This rigorous approach allowed us to assess shifts in the disease’s impact over more than 3 decades, highlighting critical changes in its prevalence and fatality rates across different regions. In addition to analyzing historical data, we also aimed to estimate the most up-to-date disability-adjusted life years (DALYs) attributable to COPD. Furthermore, we projected trends in these metrics over the next 10 years by applying the average annual percentage change (AAPC) methodology. This dual focus on current and predictive analytics serves to enhance our understanding of COPD’s burden on global health. The ultimate objective of our study is to offer a detailed evaluation of the global status of COPD, alongside its regional distribution characteristics. By furnishing these data, we hope to establish a scientific foundation that can guide public health officials and policymakers in crafting effective prevention and control strategies. This research not only fills a critical void in the literature but also aims to facilitate informed decisions that could lead to improved health outcomes for individuals affected by COPD worldwide.

## 2. Methods

This investigation encompasses several crucial elements: first, an in-depth examination and description of the epidemiological status of COPD at global, regional, and national levels; second, a thorough analysis of trends, integrating extensive, partial, and multifaceted perspectives to achieve a deeper understanding; third, an analytical synthesis that considers the effects of various factors, such as global population growth, aging demographics, and shifts in epidemiology; a comprehensive decomposition analysis of COPD incidence, mortality, and DALYs was conducted; fourth, an additional discussion illustrates the influence of COPD-related risk factors on mortality and DALYs, expressed as percentages. This specific analysis includes not only global figures but also 5 distinct Sociodemographic Index (SDI) regions (high SDI, high-middle SDI, middle SDI, low-middle SDI, and low SDI), utilizing variables such as mortality rates and DALY percentages; and finally, a predictive analysis was performed to assess the potential future effects of COPD. This research aims to thoroughly evaluate the developmental trends of global COPD prevalence and its anticipated future changes through a multitiered analytical framework, thereby establishing a robust theoretical basis for the development of public health strategies and the effective allocation of resources.

### 2.1. Data source

This research utilized data from the 2021 GBD, which encompasses 371 diseases and 88 risk factors across 204 nations and regions. This extensive dataset supports research efforts, enabling a more precise understanding of global health conditions. To maintain the integrity and accuracy of the data, we incorporated several data sources and designated appropriate identifiers, ultimately consolidating them into the Global Health Data Exchange (https://ghdx.healthdata.org). In our data processing, we employed a spatiotemporal Gaussian process regression model to address data gaps and applied smoothing adjustments to enhance the reliability of our results. The primary estimation technique utilized was the Bayesian meta-regression model (DisMod-MRV.2.1), which facilitates effective analysis in the face of complex datasets. This study focuses on COPD, emphasizing derived estimates related to its prevalence, mortality, and DALYs, while also considering the associated uncertainty intervals. This investigation is vital for understanding the global health burden associated with COPD and serves as a significant foundation for developing relevant public health policies. Furthermore, we utilized the SDI to assess the sociodemographic development levels of various countries or regions, subsequently examining the correlation between health outcomes and socioeconomic progress. In conclusion, this study leveraged GBD 2021 data to conduct a thorough evaluation of COPD, along with an analysis of sociodemographic indices aimed at uncovering the relationship between health conditions across different countries or regions and their respective social development levels. Such investigations not only aid in public health policy formulation but also offer valuable insights for future research endeavors.

### 2.2. Case definition

COPD is a condition explicitly identified by the International Classification of Diseases, 11th Revision. It is prevalent, preventable, and can be effectively treated. COPD is characterized by persistent airflow restriction that typically worsens over time, accompanied by a chronic inflammatory response triggered by harmful substances or gases. Furthermore, exacerbations and related health issues in patients with COPD significantly impact their overall well-being, complicating and exacerbating the condition. In the International Classification of Diseases, 11th Revision, COPD is classified under the code CA22, highlighting its importance in medical categorization. Additionally, within the GBD etiological classification, COPD is ranked at level 3, underscoring the substantial challenge it poses to global health.

### 2.3. Data management

The data management for this study centers on COPD and spans 4 key indicators – incidence, prevalence, mortality, and DALYs – from 1990 to 2021 at global, regional, and national levels. First, the age-standardized incidence rate (ASIR), age-standardized prevalence rate (ASPR), age-standardized mortality rate (ASMR), and age-standardized DALY rate (ASDR) were extracted from the GBD Study 2021 and stratified by sex and 5-year age groups (0–4 to 95–99 years). Absolute numbers, percentage changes, and temporal curves were calculated to characterize the spatiotemporal heterogeneity of disease burden. Pearson correlation was then used to examine the linear relationship between the SDI and each of the 4 metrics, revealing how socioeconomic development influences COPD outcomes. To disentangle the drivers of change, a decomposition analysis partitioned the changes from 1990 to 2021 into population aging, population growth, and epidemiological transition (incidence or case-fatality rate dynamics), thereby quantifying the independent contribution of each factor to incidence, prevalence, mortality, and DALYs.

Building on this foundation, we further incorporated risk-factor exposure data. Eight Level-1 risk factors – ambient particulate matter, high temperature, household air pollution, low temperature, occupational particulate matter, ozone, secondhand smoke, and active smoking – were extracted from the GBD 2021 Risk Factors database, together with their annual population attributable fractions and corresponding DALYs and deaths for the period from 1990 to 2021. Each factor was organized by SDI quintile (low, low-middle, middle, high-middle, and high) and at the global level and was further stratified by 5-year age groups and sex to ensure dimensional alignment with the COPD outcome data.

Trend analyses integrated 4 complementary statistical models. First, an age-period-cohort (APC) model, implemented in the Epi package under a Poisson framework, decomposed rate ratios by birth cohort, calendar period, and age group. Second, a Bayesian age-period-cohort (BAPC) model, run with the INLA package, integrated multidimensional uncertainty, smoothed random fluctuations, and projected COPD burden from 2022 to 2050. Third, the Joinpoint Regression Program 5.2.0 (National Cancer Institute [NCI]) was used to identify 0 to 5 optimal inflection points via grid search and Monte Carlo permutation tests, estimating the AAPC and its 95% confidence interval to classify trends as rising, falling, or stable (α = 0.05). Fourth, traditional log-linear regression provided the estimated annual percentage change as an additional check on the robustness of long-term trends. Rather than treating these models as competitors, we regarded them as complementary analytical lenses; therefore, AIC, BIC, or pseudo-*R*^2^ were not employed for model selection. All statistical procedures were conducted in R 4.4.1 (R Core Team).

## 3. Results

### 3.1. COPD burden at global and regional levels

**Figure 1.**
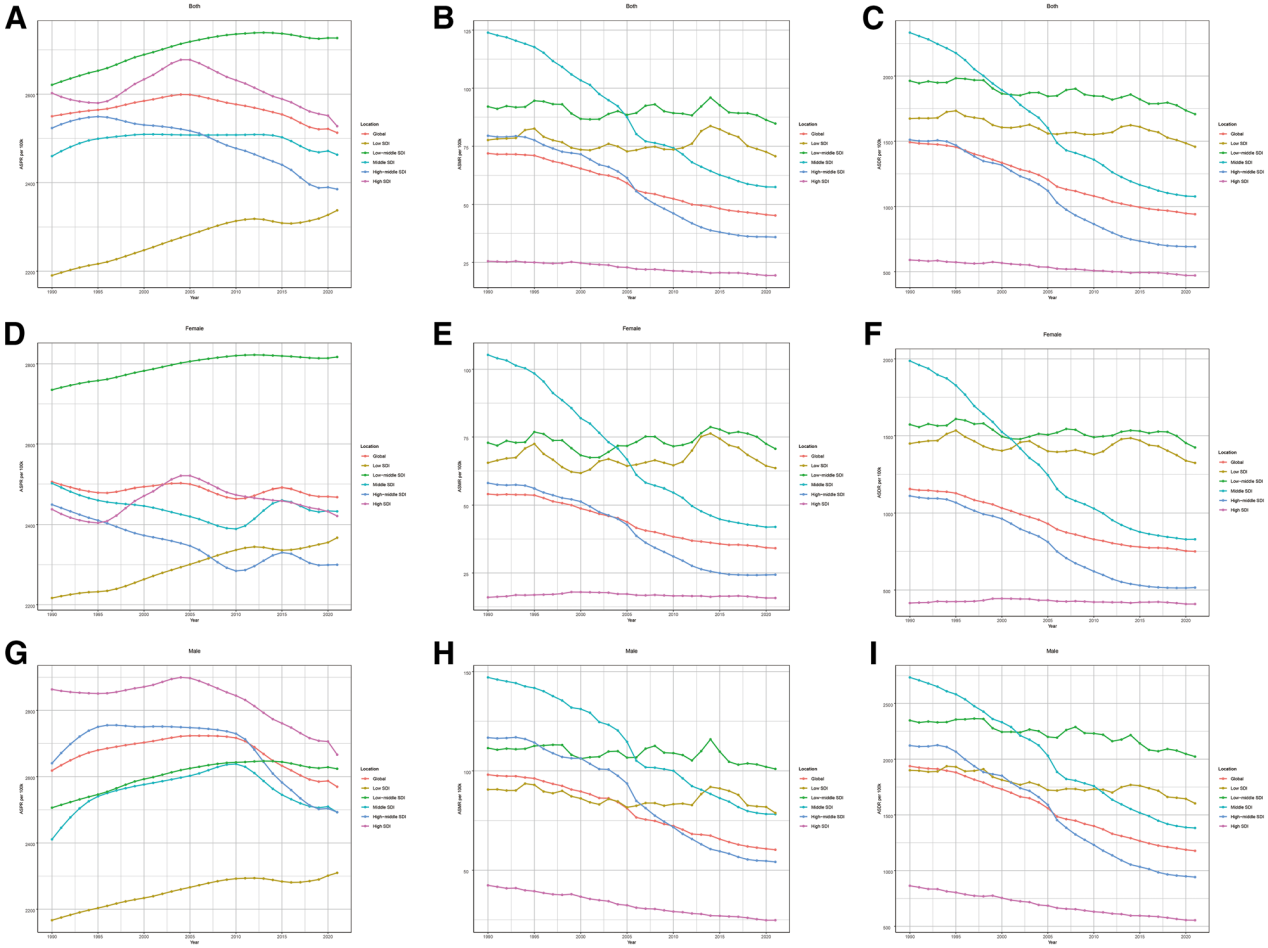
Global COPD ASR from 1990 to 2021. (A) ASPR; (B) ASMR; (C) ASDR; (D) ASPR for females; (E) ASMR for females; (F) ASDR for females; (G) ASPR for males; (H) ASMR for males; (I) ASDR for males. ASDR = age-standardized disability-adjusted life years rate, ASMR = age-standardized mortality rate, ASPR = age-standardized prevalence rate, ASR = age-standardized rate, COPD = chronic obstructive pulmonary disease.

In a global analysis of 204 countries and various regions, we examined trends in ASIR, ASPR, ASMR, and ASDR associated with COPD from 1990 to 2021. During this period, ASIR exhibited an upward trend from 1990 to 2005, followed by a downward trend from 2005 to 2021 (Fig. 1A). The overall trends in ASMR and ASDR demonstrated a continuous decline (Fig. 1B and C), which was evident in both male and female groups (Fig. 1D–I). Notably, all values for the percentage change (PC), AAPC, and estimated annual percentage change for age-standardized rates (ASRs) between 1990 and 2021 were below 0, indicating a sustained downward trend (Tables 1–4). Comparing the data from 1990 to 2021, we observed a significant increase in the number of cases, deaths, and DALYs (Tables 1–4). Additionally, the ASR values in the low-medium SDI region were significantly higher than those in other regions (Fig. 1A–C). ASIR in the low-SDI and low-medium SDI regions exhibited an upward trend, while ASMR and ASDR fluctuated during this period, showing a slight upward trend (Fig. 1B and C). Conversely, ASR in the high-SDI area demonstrated a steady downward trend (Fig. 1A–C).

**Table 1:** The number of incidence and ASIR for COPD in 1990 and 2021, and changes from 1990 to 2021.

Item	1990		2021		1990–2021		
	Number	ASIR per 100,000	Number	ASIR per 100,000	Percentage changes, %	AAPC, %	EAPC, %
Global	8,054,864.56 (7,419,999.35–8,706,820.30)	202.43 (186.70–218.25)	16,895,444.79 (15,471,347.31–18,335,690.52)	197.37 (181.65–213.42)	−0.02 (−0.05 to −0.01)	−0.08 (−0.10 to −0.07)	−0.11 (−0.13 to −0.09)
Sex							
Male	4,017,807.17 (3,719,865.45–4,325,984.04)	222.14 (205.68–238.72)	8,395,761.08 (7,714,087.18–9,116,517.46)	211.14 (195.59–227.61)	−0.05 (−0.07 to −0.03)	−0.16 (−0.19 to −0.14)	−0.21 (−0.25 to −0.17)
Female	4,037,057.39 (3,706,658.94–4,382,018.14)	187.85 (172.81–203.47)	8,499,683.71 (7,776,489.29–9,241,204.91)	185.94 (170.31–201.97)	−0.01 (−0.03 to 0.01)	−0.03 (−0.04 to −0.02)	−0.04 (−0.06 to −0.02)
SDI*							
Low	426,069.42 (394,701.57–457,628.55)	183.69 (171.24–195.93)	1,003,161.23 (931,213.77–1,080,774.13)	188.01 (174.28–201.15)	0.02 (−0.00 to 0.05)	0.07 (0.06–0.09)	0.04 (0.03–0.06)
Low-middle	1,399,413.59 (1,306,143.72–1,492,917.12)	229.77 (214.82–244.72)	3,238,337.55 (2,999,595.01–3,483,041.98)	227.24 (212.55–242.96)	−0.01 (−0.03 to 0.01)	−0.04 (−0.05 to −0.03)	−0.06 (−0.07 to −0.04)
Middle	2,233,088.08 (2,061,017.59–2,412,492.13)	217.65 (200.28–234.71)	5,330,087.72 (4,819,505.42–5,856,107.18)	203.26 (185.58–221.23)	−0.07 (−0.10 to −0.04)	−0.22 (−0.24 to −0.20)	−0.28 (−0.30 to −0.26)
High-middle	1,982,989.89 (1,822,504.91–2,159,755.71)	202.55 (186.43–219.78)	3,572,747.46 (3,232,637.57–3,922,372.55)	184.66 (168.23–202.63)	−0.09 (−0.12 to −0.06)	−0.30 (−0.33 to −0.27)	−0.39 (−0.41 to −0.36)
High	2,006,290.86 (1,813,789.39–2,192,517.40)	184.23 (166.85–200.81)	3,738,604.77 (3,455,740.61–4,029,190.31)	187.56 (174.46–200.88)	0.02 (−0.01 to 0.05)	0.07 (0.05–0.08)	0.13 (0.08–0.18)

Data in parentheses are 95% uncertainty intervals.

AAPC = average annual percentage change, ASIR = age-standardized incidence rate, COPD = chronic obstructive pulmonary disease, EAPC = estimated annual percentage change, SDI = Sociodemographic Index.

* *P* < .05.

**Table 2:** The number of ASPR for COPD in 1990 and 2021, and changes from 1990 to 2021.

Item	1990		2021		1990–2021		
	Number	ASPR per 100,000	Number	ASPR per 100,000	Percentage changes, %	AAPC, %	EAPC, %
Global	100,544,855.40 (91,213,114.61–110,608,178.60)	2550.02 (2318.34–2806.32)	213,387,446.20 (194,868,122.10–233,975,904.60)	2512.86 (2293.93–2748.52)	−0.05 (−0.07 to −0.03)	−0.05 (−0.07 to −0.03)	−0.04 (−0.08 to −0.01)
Sex							
Male	46,928,918.98 (42,428,874.04–51,554,601.64)	2618.46 (2379.32–2877.85)	100,645,850.00 (91,907,000.86–110,408,399.40)	2569.60 (2347.97–2807.82)	−0.02 (−0.04 to 0.00)	−0.06 (−0.08 to −0.04)	−0.08 (−0.14 to −0.01)
Female	53,615,936.46 (48,585,471.65–58,944,830.18)	2505.74 (2272.70–2758.88)	112,741,596.20 (102,454,382.50–123,555,027.20)	2468.19 (2244.96–2703.95)	−0.01 (−0.03 to 0.00)	−0.05 (−0.06 to −0.03)	−0.03 (−0.04 to −0.01)
SDI*							
Low	5,252,443.51 (4,737,593.52–5,757,399.66)	2190.49 (1969.32–2409.53)	12,666,829.95 (11,450,844.58–13,814,461.88)	2337.52 (2119.39–2567.85)	0.21 (0.19–0.22)	0.21 (0.19–0.22)	0.21 (0.19–0.23)
Low-middle	16,242,733.92 (14,746,584.60–17,759,305.37)	2621.01 (2378.69–2867.91)	38,692,639.02 (35,061,911.35–42,291,464.22)	2726.75 (2478.22–2979.00)	0.13 (0.12–0.14)	0.13 (0.12–0.14)	0.14 (0.11–0.16)
Middle	25,869,096.45 (23,297,858.38–28,419,526.14)	2459.97 (2217.17–2717.88)	63,927,638.63 (57,320,694.57–71,316,409.59)	2463.19 (2208.84–2735.96)	0.00 (−0.01 to 0.02)	0.00 (−0.02 to −0.01)	−0.01 (−0.03 to 0.02)
High-middle	24,588,397.98 (22,221,499.96–27,193,555.86)	2523.42 (2287.88–2794.38)	45,671,522.59 (40,973,796.19–50,846,209.89)	2385.34 (2149.82–2648.74)	−0.18 (−0.20 to −0.16)	−0.18 (−0.20 to −0.16)	−0.22 (−0.25 to −0.19)
High	28,494,604.80 (25,831,583.24–31,241,950.34)	2602.27 (2359.28–2847.55)	52,256,394.66 (48,386,829.86–55,979,572.19)	2527.66 (2364.40–2701.80)	−0.08 (−0.10 to −0.06)	−0.08 (−0.10 to −0.06)	−0.04 (−0.1 to 0.02)

Data in parentheses are 95% uncertainty intervals.

AAPC = average annual percentage change, ASPR = age-standardized prevalence rate, COPD = chronic obstructive pulmonary disease, EAPC = estimated annual percentage change, SDI = Sociodemographic Index.

* *P* < .05.

**Table 3:** The number of mortality and ASMR for COPD in 1990 and 2021, and changes from 1990 to 2021.

Item	1990		2021		1990–2021		
	Number	ASMR per 100,000	Number	ASMR per 100,000	Percentage changes, %	AAPC, %	EAPC, %
Global	2,495,513.45 (2,238,986.25–2,694,755.05)	71.92 (64.47–77.53)	3,719,936.55 (3,347,912.32–4,084,218.20)	45.22 (40.61–49.70)	−0.37 (−0.43 to −0.28)	−1.49 (−1.61 to −1.36)	−1.75 (−1.84 to −1.65)
Sex							
Male	1,417,119.70 (1,268,504.16–1,563,697.95)	98.09 (87.78–107.55)	2,120,474.44 (1,837,654.01–2,329,589.63)	60.30 (52.45–66.10)	−0.39 (−0.48 to −0.30)	−1.63 (−1.81 to −1.44)	−1.79 (−1.88 to −1.70)
Female	1,078,393.75 (846,631.97–1,217,505.07)	54.08 (42.64–60.88)	1,599,462.12 (1,355,122.78–1,856,873.09)	34.12 (28.92–39.62)	−0.37 (−0.47 to −0.18)	−1.47 (−1.57 to −1.36)	−1.77 (−1.88 to −1.66)
SDI*							
Low	133,099.95 (106,538.23–156,186.18)	77.67 (61.91–91.44)	266,003.52 (237,374.65–300,954.63)	70.70 (63.35–79.76)	−0.09 (−0.20 to 0.12)	−0.29 (−0.70 to 0.13)	−0.11 (−0.27 to 0.05)
Low-middle	443,218.09 (352,861.50–517,320.13)	92.07 (74.17–107.46)	999,762.37 (898,211.09–1,104,472.17)	84.76 (75.80–93.78)	−0.08 (−0.20 to 0.16)	−0.23 (−0.77 to 0.31)	−0.12 (−0.23 to −0.02)
Middle	954,485.27 (837,573.01–1,044,246.29)	123.89 (109.19–134.80)	1,293,660.10 (1,122,165.50–1,476,736.57)	57.45 (49.59–65.43)	−0.54 (−0.60 to −0.44)	−2.51 (−2.72 to −2.29)	−2.85 (−2.98 to −2.72)
High-middle	673,578.21 (599,623.03–733,445.51)	79.53 (70.61–86.61)	689,449.01 (591,990.84–781,694.40)	35.91 (30.78–40.69)	−0.55 (−0.62 to −0.47)	−2.57 (−2.78 to −2.36)	−3.15 (−3.39 to −2.91)
High	289,771.17 (270,268.54–300,481.60)	25.53 (23.71–26.50)	469,238.51 (410,389.19–501,052.68)	19.44 (17.26–20.66)	−0.24 (−0.28 to −0.21)	−0.86 (−1.01 to −0.72)	−0.97 (−1.04 to −0.90)

Data in parentheses are 95% uncertainty intervals.

AAPC = average annual percentage change, ASMR = age-standardized mortality rate, COPD = chronic obstructive pulmonary disease, EAPC = estimated annual percentage change, SDI = Sociodemographic Index.

* *P* < .05.

**Table 4:** The number of DALYs and ASDR for COPD in 1990 and 2021, and changes from 1990 to 2021.

Item	1990		2021		1990–2021		
	Number	DALYs per 100,000	Number	DALYs per 100,000	Percentage changes, %	AAPC, %	EAPC, %
Global	56,857,290.42 (51,293,861.39–61,412,141.62)	1492.64 (1342.46–1609.30)	79,779,695.05 (74,026,373.40–86,011,405.86)	940.65 (871.48–1014.59)	−0.37 (−0.42 to −0.29)	−1.48 (−1.54 to −1.42)	−1.71 (−1.79 to −1.63)
Sex							
Male	32,569,411.80 (29,042,990.70–35,889,346.27)	1937.66 (1737.82–2127.01)	45,057,823.53 (39,954,987.88–49,273,235.21)	1177.27 (1047.92–1288.56)	−0.39 (−0.47 to −0.31)	−1.59 (−1.71 to −1.47)	−1.82 (−1.89 to −1.74)
Female	24,287,878.62 (19,631,542.37–27,341,372.05)	1155.29 (934.32–1299.04)	34,721,871.52 (30,574,577.72–39,278,826.24)	750.60 (661.98–848.24)	−0.35 (−0.43 to −0.18)	−1.39 (−1.49 to −1.29)	−1.63 (−1.73 to −1.54)
SDI*							
Low	3,510,887.75 (2,843,503.04–4,053,896.08)	1673.81 (1373.37–1936.98)	6,693,611.03 (6,035,071.60–7,482,882.34)	1457.94 (1318.76–1617.04)	−0.13 (−0.22 to 0.05)	−0.42 (−0.68 to −0.15)	−0.38 (−0.46 to −0.30)
Low-middle	11,127,270.97 (8,989,711.90–12,759,941.55)	1963.20 (1602.24–2252.75)	22,727,337.53 (20,885,631.75–24,793,577.27)	1707.90 (1558.88–1865.11)	−0.13 (−0.24 to 0.07)	−0.39 (−0.81 to 0.03)	−0.38 (−0.44 to −0.32)
Middle	21,436,985.15 (18,993,061.55–23,538,972.62)	2332.91 (2063.49–2546.31)	26,679,162.31 (23,958,688.03–29,735,634.14)	1076.67 (963.62–1201.24)	−0.54 (−0.60 to −0.46)	−2.52 (−2.68 to −2.35)	−2.83 (−2.94 to −2.72)
High-middle	14,132,282.31 (12,756,902.61–15,327,841.39)	1511.32 (1365.74–1635.67)	13,439,404.01 (12,068,466.43–15,035,539.57)	691.14 (621.83–772.74)	−0.54 (−0.60 to −0.47)	−2.53 (−2.71 to −2.35)	−3.06 (−3.25 to −2.87)
High	6,617,361.45 (6,259,951.10–6,910,462.60)	589.80 (557.84–616.39)	10,197,032.65 (9,399,334.70–10,811,522.80)	471.22 (437.45–498.84)	−0.20 (−0.22 to −0.18)	−0.72 (−0.89 to −0.54)	−0.76 (−0.80 to −0.71)

Data in parentheses are 95% uncertainty intervals.

AAPC = average annual percentage change, ASDR = age-standardized DALYs rate, COPD = chronic obstructive pulmonary disease, DALYs = disability-adjusted life years, EAPC = estimated annual percentage change, SDI = Sociodemographic Index.

* *P* < .05.

Globally, the prevalence of COPD increased significantly, rising from 100,544,855.40 cases in 1990 to 213,387,446.20 cases in 2021. During this period, the mean annual percentage change in ASIR was −0.05, indicating a downward trend (Table 2). Similarly, the number of age-standardized deaths due to COPD increased from 80,548,864.56 in 1990 to 1,689,544,479.00 in 2021, while the ASMR decreased from 71.29 per 100,000 in 1990 to 45.22 per 100,000 in 2021, with an AAPC of −1.49, marking the most significant decline (Table 3). Concurrently, COPD-related DALYs decreased from 1492.64 per 100,000 in 1990 to 940.65 per 100,000 in 2021, and the AAPC of the ASDR was −1.48, demonstrating a clear downward trend (Table 4).

**Figure 2.**
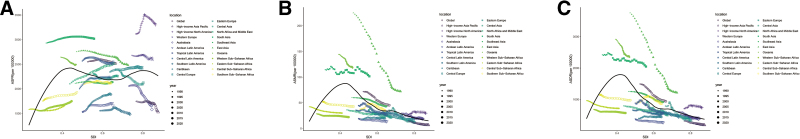
The relationship between COPD ASPR, ASMR, ASDR, and SDI. (A) The relationship between ASPR and SDI across 21 regions from 1990 to 2021. (B) The relationship between ASMR and SDI across 21 regions from 1990 to 2021. (C) The relationship between ASDR and SDI across 21 regions from 1990 to 2021. ASDR = age-standardized disability-adjusted life years rate, ASMR = age-standardized mortality rate, ASPR = age-standardized prevalence rate, COPD = chronic obstructive pulmonary disease, SDI = Sociodemographic Index.

The overall ASPR, ASMR, and ASDR for COPD exhibited a downward trend with increasing SDI (Fig. 2A–C). Our study further revealed an inverted V-shaped association between ASPR, ASMR, and SDI in the age-standardized incidence of COPD from 1990 to 2021. Data from most regions indicated that the ASR increased exponentially with the Sociodemographic Index (SDI) until it began to decline when the SDI reached approximately 0.4. At this threshold, the ASPR started to decrease, and with the continuous improvement of SDI, the ASPR index rose again, only to decline once more with further increases in SDI (Fig. 2A). For ASMR and ASDR, the initial rise in SDI triggered a simultaneous increase in these 2 indicators, followed by a subsequent decline (Fig. 2B and C). Notably, the ASPR values in the high-income North America region were significantly higher than those in other regions, while the ASMR and ASDR in the East Asia region were markedly elevated compared with those in other areas (Fig. 2A–C).

### 3.2. Decomposition analysis of ASR

**Figure 3.**
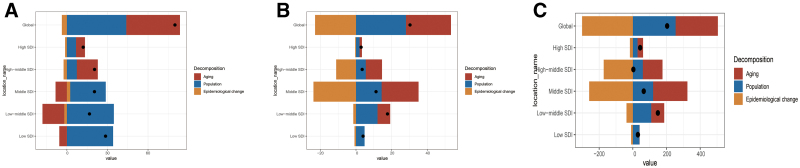
Changes in the global burden of COPD from 1990 to 2021, stratified by SDI quintiles: a population-level determinants analysis based on population size, aging, and epidemiological changes. (A) Changes in global and SDI quintile-stratified COPD incidence from 1990 to 2021, according to population-level determinants of population size, aging, and epidemiological changes. (B) Changes in global and SDI quintile-stratified COPD deaths from 1990 to 2021, according to population-level determinants of population size, aging, and epidemiological changes. (C) Changes in global and SDI quintile-specific COPD DALYs from 1990 to 2021 based on population-level determinants including population size, aging, and epidemiological shifts. COPD = chronic obstructive pulmonary disease, DALYs = disability-adjusted life years, SDI = Sociodemographic Index.

Figure 3 illustrates the COPD ASR across various SDI regions from 1990 to 2021. Globally, factors such as aging and population size positively influenced the incidence, mortality, and disability rates of COPD, particularly in regions with middle and low SDI. While epidemiological changes had a negative impact (as shown in Fig. 3A–C), their effects have been relatively consistent across different SDI regions. From 1990 to 2021, the population base factor of COPD ASIR accounted for a substantial portion (54.96%), with the highest increase observed in the SDI regions. Although epidemiological changes negatively affected all 5 SDI regions, their impact in low-SDI areas was minimal, contributing only 0.09% (see Fig. 3A). Overall, the population base factor for COPD ASMR constituted a significant proportion (92.4%), with the highest increase noted in high-SDI areas. In contrast, both population aging and epidemiological changes negatively impacted low-SDI areas, accounting for 0.75% and 20.41%, respectively (refer to Fig. 3B). From a global perspective, the population base factor for COPD DALYs also represented a considerable proportion. In low-SDI regions, the contribution of aging factors to COPD DALYs was the smallest (3.69%), whereas in high-SDI regions, aging factors comprised the largest proportion of COPD DALYs (see Fig. 3C).

### 3.3. COPD burden attributable to risk factors

**Figure 4.**
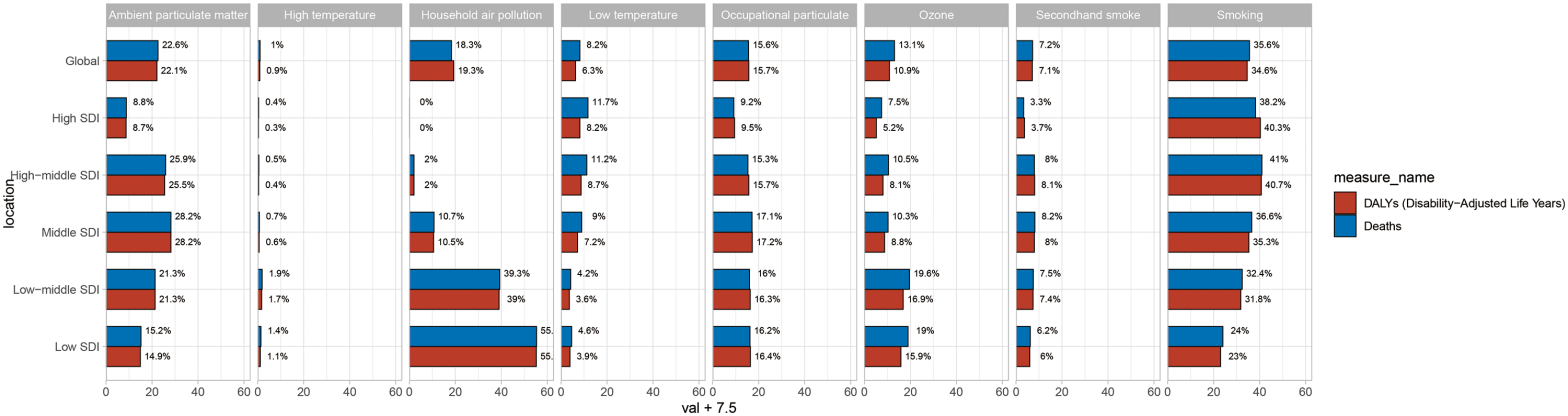
The proportion of the impact of COPD-related risk factors on DALYs and deaths across different SDI regions and globally, 1990 to 2021. COPD = chronic obstructive pulmonary disease, DALYs = disability-adjusted life years, SDI = Sociodemographic Index.

Figure 4 depicts the effects of various risk factors on DALYs and mortality associated with COPD in different SDI regions, as well as on a global scale, from 1990 to 2021. The GBD 2021 study identified several key risk factors that affected DALYs and mortality rates due to COPD, including ambient particulate matter, elevated temperatures, household air pollution, cold temperatures, occupational particulate matter, ozone levels, secondhand smoke exposure, and smoking habits. The impact of these risk factors on COPD differed between various SDI regions and on a global level. On a global scale, the primary risk factors contributing to DALYs and deaths related to COPD include smoking, pollution from ambient particulate matter, and indoor air pollution, with high temperatures exerting the least influence (DALYs = 0.9%, deaths = 1.0%). There are notable variations across different regions in terms of the burden associated with COPD and its key risk factors. In regions with a low SDI, indoor air pollution has the greatest effect on both DALYs and fatalities (DALYs = 55.0%, deaths = 55.0%), whereas high temperatures show a relatively minor effect (DALYs = 1.4%, deaths = 1.1%). Similarly, in low-middle-SDI regions, the impact of indoor air pollution on DALYs and death rates is significant (DALYs = 39.3%, deaths = 39.0%). In regions with a middle SDI, smoking has a notable impact on DALYs and mortality, contributing to 36.6% and 35.3%, respectively. The effect of smoking is even more considerable in high-middle-SDI areas, where it accounts for 41.0% of DALYs and 40.7% of mortality. In regions classified as high SDI, smoking maintains a significant influence on DALYs and mortality, with rates of 38.2% and 40.3%, respectively. On the other hand, indoor air pollution seems to exert minimal effects on both DALYs and mortality, yielding a rate of 0%. When focusing on particular risk factors, global pollution from ambient particulate matter is a key driver of DALYs and mortality, especially within low-middle- and middle-SDI regions. The effects of elevated temperatures on DALYs and mortality are relatively limited worldwide, although they are more substantial in low- and low-middle-SDI regions in comparison to high-, high-middle-, and middle-SDI regions. Indoor air pollution stands out as the most significant global factor, specifically affecting middle- and high-middle-SDI areas. While the influence of low temperatures on DALYs and mortality is generally minor worldwide, its mortality impact is significant in high- and high-middle-SDI regions. The global effect of occupational particulate matter on DALYs and mortality is restricted, though it holds greater significance in low-middle- and high-middle-SDI contexts. Similarly, the impact of ozone on DALYs and mortality is minor at a global level but exhibits notable effects in low- and low-middle-SDI regions. Secondhand smoke appears to have a minimal effect on DALYs and mortality on a global scale, yet its influence is more significant in high-middle- and middle-SDI areas. In summary, while smoking’s impact on DALYs and mortality is considerable on a global scale, it is less significant in low-SDI regions (Fig. 4).

### 3.4. COPD burden at the country level

**Figure 5.**
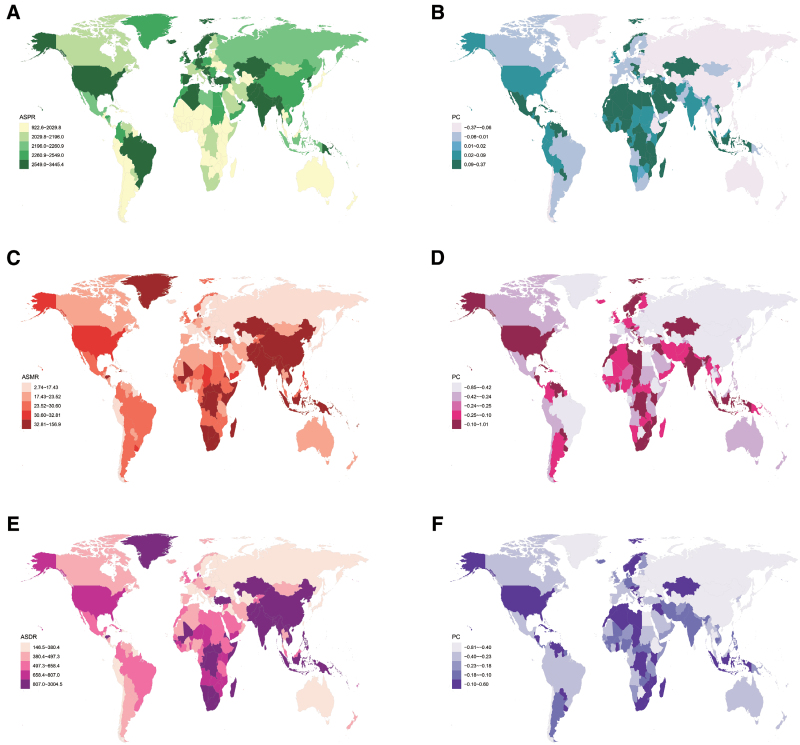
COPD Global Burden 1990 to 2021: ASPR, ASMR, and ASDR with PC. (A) COPD ASPR in 2021; (B) PC of the standardized COPD ASPR from 1990 to 2021; (C) COPD ASMR in 2021; (D) PC of the standardized COPD ASMR from 1990 to 2021; (E) COPD ASDR in 2021; (F) PC of the standardized COPD ASDR from 1990 to 2021. ASDR = age-standardized disability-adjusted life years rate, ASMR = age-standardized mortality rate, ASPR = age-standardized prevalence rate, COPD = chronic obstructive pulmonary disease, PC = percent change.

The burden of COPD showed notable variations across the globe from 1990 to 2021, with significant differences observed among countries. A comprehensive analysis of the COPD burden in 2021, which included data from 204 countries and regions, revealed that certain countries, such as the Republic of the Union of Myanmar, the Solomon Islands, and Turkmenistan, had markedly higher COPD prevalence rates than other countries. In contrast, the Republic of Sudan reported the lowest prevalence figures among the assessed countries, highlighting regional disparities in the disease’s impact (Fig. 5A–C). Additionally, the regions exhibiting elevated ASPR were largely concentrated in specific countries across South America, North Africa, the Middle East, and South Asia. This pattern suggests not only the geographic distribution of health burdens but also the varying access to healthcare resources and environmental factors affecting these regions. Over the course of 3 decades, from 1990 to 2021, many parts of Africa, South Asia, Southeast Asia, South America, and the Middle East recorded high prevalence coefficient values, indicating a concerning upward trend in ASPR among these areas. Conversely, regions such as North America and Europe, along with some others, displayed relatively lower PC values, with some areas even experiencing negative growth rates. This decline points to a possible improvement in health outcomes or effective interventions for COPD in those regions. For instance, the PC prevalence in Mongolia was 0.37, while the Federated States of Micronesia and the Republic of the Philippines had PC values of 0.35 and 0.32, respectively, all of which were significantly higher than those in many other countries. In the African region, the distribution of PC values was particularly varied, ranging from negative to positive values. This diversity underscores the existence of considerable numerical differences among various nations, where some countries may be experiencing a rising trend in COPD prevalence while others are witnessing a decline. Although there was an observable upward trend in PC within the African region, the overall ASPR did not reflect a high prevalence rate, suggesting complex underlying factors influencing the burden of COPD in this area (Fig. 5A and D).

In 2021, the distribution of COPD mortality rates revealed significant geographical disparities. The regions experiencing the highest mortality rates from COPD were primarily located in South Asia, Southeast Asia, and Central Africa. Conversely, the areas that reported lower mortality rates were predominantly found in North America, Australia, New Zealand, and various European nations. Notably, Swaziland exhibited a substantially elevated COPD mortality rate compared with other regions, whereas Sudan was distinguished by having the lowest mortality rate for this disease, as illustrated in Figure 5C. Over the period from 1990 to 2021, there was a discernible trend of increasing ASMR in specific countries located in South Asia, Southeast Asia, and Central and West Africa. This rising pattern of mortality rates raised concerns regarding the overall public health implications in these regions. Furthermore, the reported mortality rates associated with pneumonia and PC were notably high in the Republic of Angola, at 1.01; the Republic of Yemen, at 0.72; and the Republic of Ecuador, at 0.58, all of which were significantly elevated compared with those in other regions. In stark contrast, the PC values in North America, Australia, New Zealand, and certain European countries were negative. This negative trend indicates a reduction in ASMRs, suggesting improvements in health outcomes and healthcare interventions in these areas (Fig. 5B and E).

In 2021, the DALYs in the Maldives and Papua New Guinea were notably elevated compared with those in other regions, indicating a significant burden of disease in these areas. Conversely, Guinea recorded the lowest DALYs, suggesting a relatively better health status or lower disease prevalence. This disparity in DALYs highlights the varying health challenges faced by different countries and regions. Furthermore, the ASDR data revealed that South Asia, Southeast Asia, as well as Central and West Africa, experienced higher ASDR rates, indicating that these regions are grappling with considerable health-related issues that adversely affect mortality. In contrast, regions such as North America, Australia, New Zealand, and various European countries reported lower ASDR rates, which may reflect their more advanced healthcare systems and better health outcomes. Between 1990 and 2021, a significant upward trend in ASDR was observed in South Asia, Southeast Asia, and Central and West Africa, as evidenced by higher values of population composition. This trend raised concerns about the escalating health burdens in these regions. On the other hand, North America, Australia, New Zealand, and certain European countries displayed lower PC values, with some even showing negative growth rates, implying improvements in their health metrics over time. Such trends suggest a divergence in health progress between more developed and less developed regions. Specifically, the DALYs per capita indicated a value of 1.01 for the Republic of Angola, 0.60 for the Republic of Indonesia, and 0.51 for the Kingdom of Cambodia. These figures were notably higher than those recorded in other regions, while simultaneously, the ASDR was increasing rapidly, underscoring the growing health challenges in these countries (Fig. 5C and F).

### 3.5. COPD burden at age and sex levels

**Figure 6.**
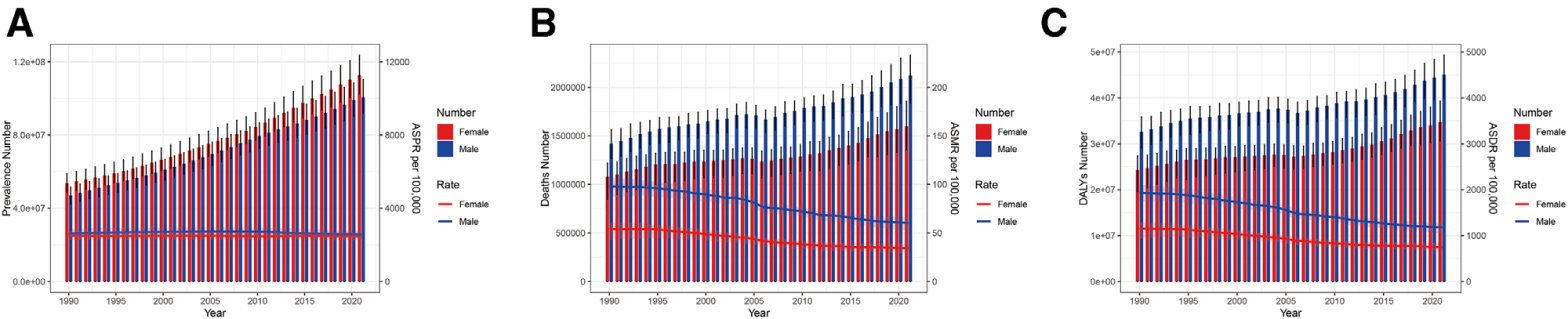
Trends in the gender-specific numbers and ASRs of COPD prevalence, deaths, and DALYs globally from 1990 to 2021. (A) Gender-specific numbers of prevalence and ASPR from 1990 to 2021; (B) gender-specific numbers of deaths and ASMR from 1990 to 2021; (C) gender-specific numbers of DALYs and ASDR from 1990 to 2021. ASDR = age-standardized disability-adjusted life years rate, ASMR = age-standardized mortality rate, ASPR = age-standardized prevalence rate, ASR = age-standardized rate, COPD = chronic obstructive pulmonary disease, DALYs = disability-adjusted life years.

Between 1990 and 2021, there was an annual rise in the incidence of COPD cases, deaths, and DALYs for both genders. While the ASPR did not demonstrate a notable upward trend, the ASMR and ASDR showed a declining pattern. Data revealed that the incidence of cases was more prevalent among females than among males, whereas males had a higher number of deaths and DALYs. Furthermore, the ASRs in males exceeded those in females (Fig. 6A–C).

**Figure 7.**
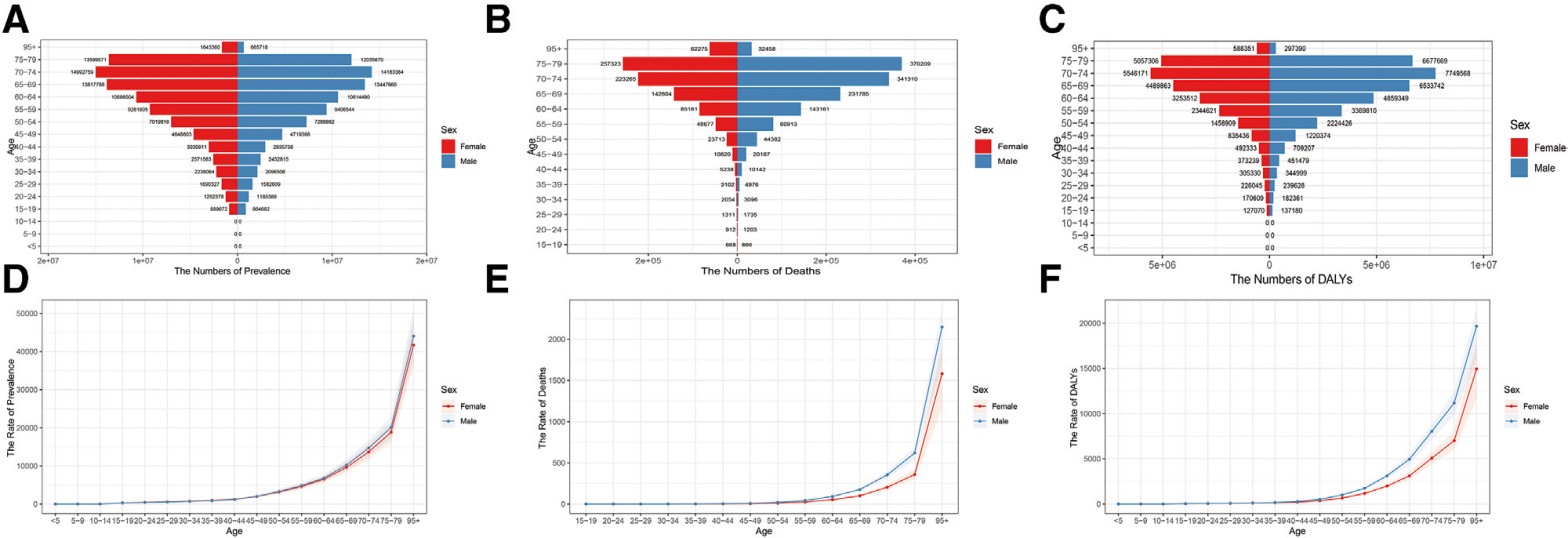
Age-specific numbers and age-standardized rates of prevalence, mortality, and DALYs for COPD. (A) Age-specific number of prevalent cases; (B) age-specific number of deaths; (C) age-specific number of DALYs; (D) age-standardized prevalence rate; (E) age-standardized mortality rate; (F) age-standardized DALYs rate. COPD = chronic obstructive pulmonary disease, DALYs = disability-adjusted life years.

Figure 7 displays the global statistics on prevalence, mortality, DALYs, and ASRs for COPD across various age groups. Mortality rates and DALY estimates were consistently higher for males than for females in every age category. Regarding prevalence, the number of cases among females surpassed that among males in the age brackets of 15 to 19, 20 to 24, 25 to 29, 30 to 34, 35 to 39, and 40 to 44 years. On the contrary, males outnumbered females in terms of cases in the age groups of 45 to 49, 50 to 54, 55 to 59, 60 to 64, 65 to 69, and 70 to 74 years (refer to Fig. 7A). Both male and female prevalence, mortality, and DALYs escalated with increasing age. For males, patient numbers did not start to decrease until reaching the age range of 70 to 74 (illustrated in Fig. 7A), while mortality figures only began to fall after the age range of 75 to 79 years (shown in Fig. 7B), and DALYs did not see a reduction until the 70 to 74 age group (depicted in Fig. 7C). Similarly, female patient numbers also did not begin to decline until the age of 70 to 74 (as indicated in Fig. 7A), death counts did not start to diminish until the age of 75 to 79 (refer to Fig. 7B), and DALYs did not decrease until the 70 to 74 age range (demonstrated in Fig. 7C). The prevalence of COPD significantly affected individuals aged 40 years and older across all categories, with ASPR, ASMR, and ASDR for men exceeding those for women. The ASPR for each age category rose steadily beyond the age of 25, with a considerable increase evident after 50 and a pronounced spike following 79 (illustrated in Fig. 7D). The ASMR progressively climbed after age 59, showing a dramatic rise post-79 years, with the increase being markedly greater in males compared with females (shown in Fig. 7E). A similar pattern was observed for ASDR, which increased steadily after age 35 years and surged sharply post-79 years (depicted in Fig. 7F).

### 3.6. Joinpoint regression analysis

**Figure 8.**
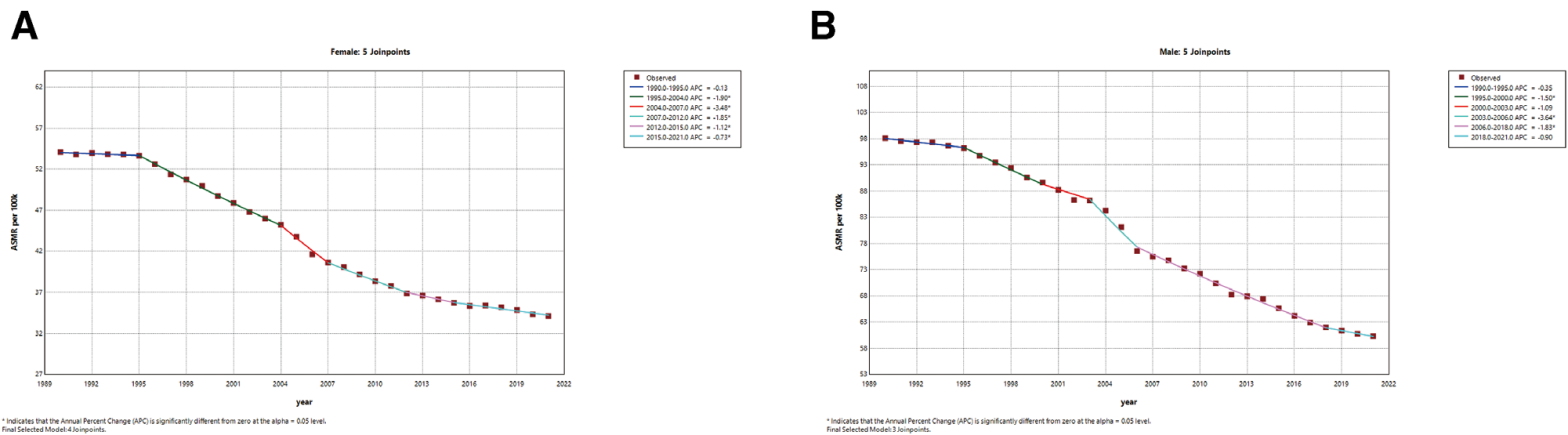
Joinpoint regression analysis of the global age-standardized mortality rate for COPD from 1990 to 2021. (A) Age-standardized mortality rate for females. (B) Age-standardized mortality rate for males. ASMR = age-standardized mortality rate, COPD = chronic obstructive pulmonary disease.

The regression analysis concerning age-standardized COPD rates in China spanning from 1990 to 2021 is illustrated in Figure 8. Our results revealed that during the period from 1990 to 1995, there was merely a minimal reduction in ASRs for both genders. In contrast, between 1995 and 2003, a notable drop in these rates for both males (1995–2000, APC = −1.50; 2000–2003, APC = −1.09) and females (1995–2000, APC = −1.90; 2000–2003, APC = −3.48) was detected (Fig. 8A). Particularly, a significant decrease in ASRs for males (2003–2006, APC = −3.64) was evident from 2003 to 2006 (Fig. 8B).

### 3.7. The effects of age, period, and cohort on incidence and mortality rates

**Figure 9.**
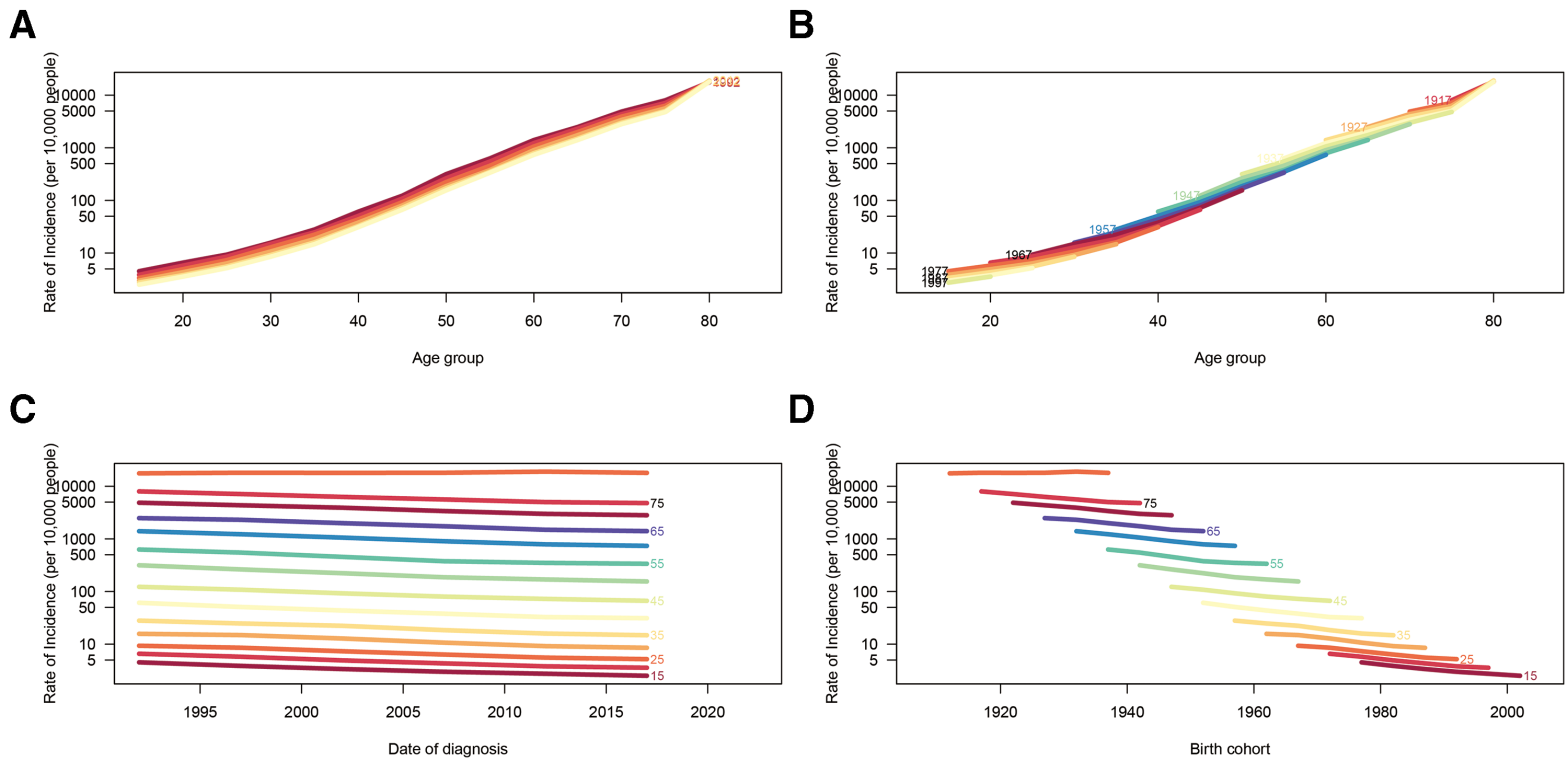
APC model analysis of COPD incidence. (A) Age-specific incidence rates of COPD segmented by time periods; each line connects the age-specific incidence rates over a 5-year period. (B) Age-specific incidence rates of COPD segmented by birth cohorts; each line connects the age-specific incidence rates of a 5-year cohort. (C) Incidence rates of COPD by age groups; each line connects the birth cohort-specific incidence rates of a 5-year age group. (D) Birth cohort-specific incidence rates of COPD segmented by different age groups; each line connects the birth cohort-specific incidence rates of a 5-year age group. APC = age-period-cohort, COPD = chronic obstructive pulmonary disease.

Figure 9A depicts the rates of incidence over time across different age brackets, with data collected from the years 1992, 1997, 2002, 2007, and 2012. The illustration reveals a trend where the incidence escalates with advancing age, especially among those aged 80 years and older. Conversely, for individuals younger than 20 years, the incidence rate remains relatively low, at roughly 5 per 100,000 individuals. Beginning at age 20 years, there is a gradual increase in the incidence rate, with the 40 to 60 age group exhibiting rates between 50 and 100 per 100,000 individuals. Importantly, the incidence rate for people over 60 years demonstrates a marked rise, reaching nearly 5000 per 100,000 individuals within the over-80 age category (Fig. 9A).

The trends in incidence rates across different age categories are depicted in Figure 9B. It is noteworthy that these rates show a steady rise over time, especially among younger cohorts. In detail, the incidence rate for individuals aged 20 to 30 years climbed from 5 cases per 100,000 people in 1967 to 100 cases per 100,000 people by 2012. Likewise, the rate for the 40 to 50 age category grew from 10 cases per 100,000 people in 1967 to 200 cases per 100,000 people by 2012 (Fig. 9B).

Figure 9C illustrates the incidence rates across different age groups from 1995 to 2020. Overall, the incidence rates for all age groups showed a consistent increase over this period. Specifically, the incidence among individuals aged 15 to 25 years rose from 5 per 100,000 in 1995 to 25 per 100,000 in 2020. Similarly, the incidence for the 45 to 55 age group increased from 50 per 100,000 in 1995 to 200 per 100,000 in 2020. Notably, the incidence for individuals aged 65 to 75 years surged from 500 per 100,000 in 1995 to 5000 per 100,000 by 2020 (Fig. 9C).

Figure 9D displays the variations in incidence rates based on particular birth years. As age increases, the incidence rates rise; however, individuals born in more recent years tend to have lower rates. The data suggest that people born in 1920 have relatively elevated incidence rates across different age categories, while those born in 2000 present notably lower rates within the same age categories. In general, there is a steady decrease in incidence rates as the birth year advances (Fig. 9D).

### 3.8. Projection of 30 years

**Figure 10.**
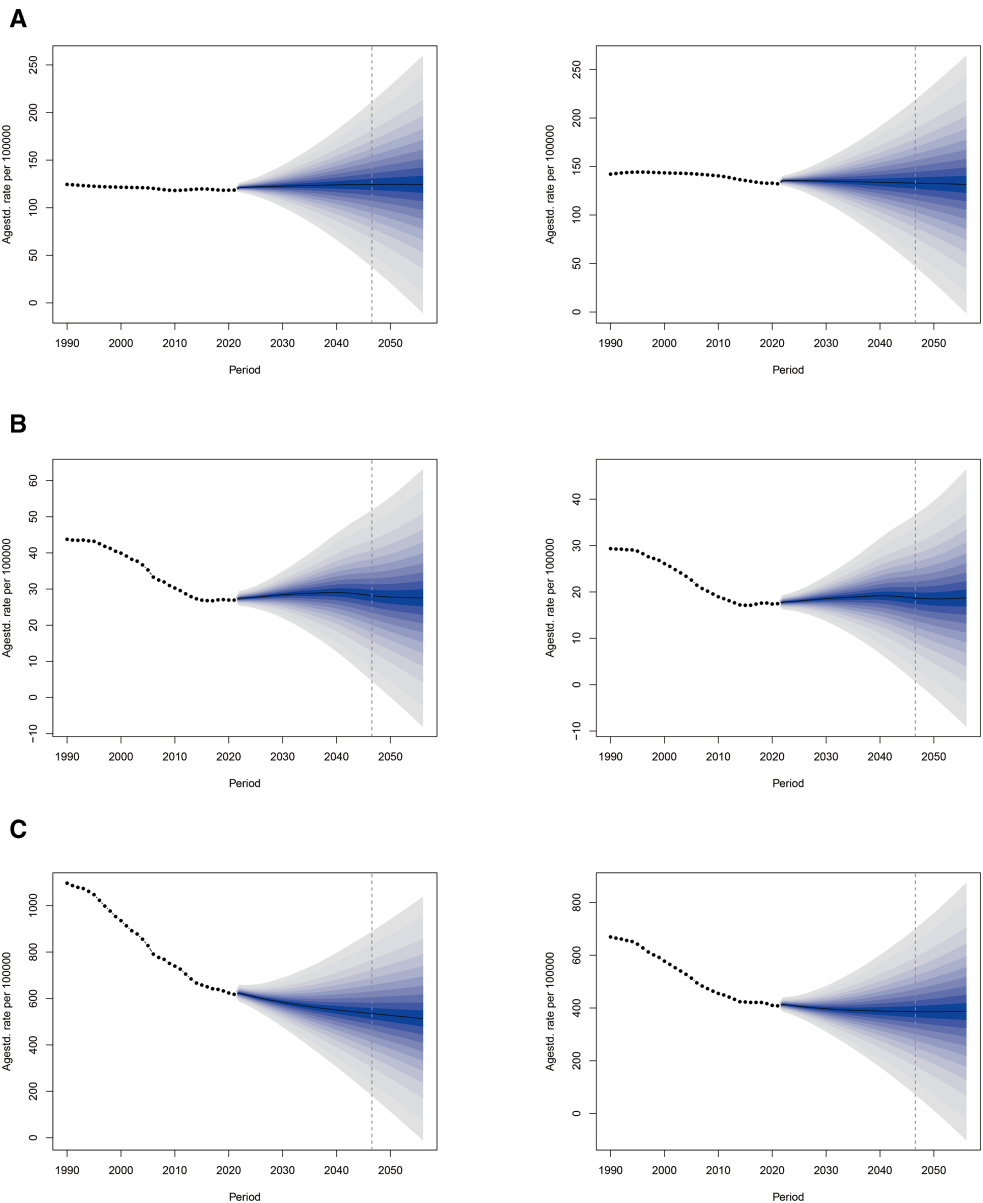
Global COPD projections for the next 30 years. (A) Gender-specific age-standardized incidence rate projections. (B) Gender-specific age-standardized mortality rate projections. (C) Gender-specific age-standardized disability-adjusted life years projections. Data presented as dashed lines in the figure represent projected scenarios, while solid lines correspond to actual observed data. COPD = chronic obstructive pulmonary disease.

To assess the trends in ASR of COPD post-2021, we utilized the BAPC model to forecast the ASR from 2021 through 2050, differentiated by gender. The results are depicted in Figure 10. We predict that over the next 30 years, the ASIR, ASMR, and ASDR associated with COPD will stabilize (see Fig. 10A–C). Notably, Figure 10A suggests that the ASIR for both men and women is projected to show stability with only slight variations throughout the upcoming 3 decades. In Figure 10B, a modest reduction in the ASMR for both sexes is indicated during this timeframe. Additionally, Figure 10C demonstrates a small decrease in the ASDR for men over the next 3 decades.

## 4. Discussion

This research utilizes the GBD 2021 database to systematically evaluate the evolving disease burden of COPD globally, regionally, and nationally from 1990 to 2021. Additionally, it applies the BAPC model to forecast prevalence trends extending to 2050. The analysis incorporates diverse methodologies, including decomposition analysis, risk factor attribution, Joinpoint regression, and the APC framework, to quantitatively examine epidemiological characteristics alongside their determinants. It further analyzes the distinct effects of population growth, aging, and prevalence shifts on the incidence, prevalence, mortality, and DALYs related to COPD. Findings indicate that although there is a persistent decline in standardized incidence, mortality, and DALY rates, the actual counts of cases, total deaths, and cumulative DALYs continue to rise. This apparent paradox is largely due to the compounded effects of an aging population and base expansion. The study highlights that COPD continues to pose a substantial challenge to global public health, particularly in nations with low-to-middle SDI. Furthermore, Zou et al reported notable increases in COPD burden linked to risk factors in countries with low and middle SDI,^[12]^ which echoes the conclusions of this study and underscores that population aging is the major contributor to the rise in absolute burden beyond 2022.

This research presents a novel finding of a unique “inverted V-shaped” relationship among the ASMR, ASPR, and ASDR of COPD in correlation with the SDI. Specifically, when the SDI falls within a low-to-middle range of 0.4 to 0.6, the burden of disease increases in tandem with economic advancement, indicating that interventions have not yet effectively addressed early industrial pollution and smoking rates. Conversely, beyond an SDI of 0.7, there is a notable decline in the curve, signifying that regions with higher SDIs have managed to counteract the initial adverse effects by investing considerable financial resources in environmental management (including ozone regulation and air quality laws^[13]^) and improving access to healthcare services (through the popularization of high-resolution computed tomography and the establishment of grassroots screening networks^[14]^). Nevertheless, the SDI alone does not fully explain all variations; the ASMR in affluent areas such as North America remains elevated compared with that in certain middle-SDI nations, implying that contemporary lifestyle elements, such as aging, the industrialization of diet, new pollutant exposures, and decreased physical activity, challenge the conventional “income-health” premise.^[15,16]^ This underscores the importance of identifying and safeguarding vulnerable groups, such as low-income elderly individuals living alone or minority populations, even in high-SDI nations.

This research identified that the ASMR in South Asia exceeds that in middle- to high-SDI nations in East Asia, while the ASPR surpasses that in East Asia across all SDI categories. A study conducted by Wang et al demonstrated that from 1990 to 2021, the yearly average variations in ASIR, ASMR, and ASDR in South Asia were +3.02%, −11.27%, and −15.47%, respectively. In contrast, in East Asia, these figures were −9.85%, −67.95%, and −67.62%, indicating a continuously widening gap between the 2 regions.^[17]^ In Indian communities, the COPD awareness rate is under 1%,^[18]^ which coincides with a culture that promotes cigarette use, weak enforcement of tobacco regulations, and limited access to smoking cessation resources.^[19]^ Rapid population aging^[20]^ further exacerbates the situation, resulting in ineffective prevention and control measures. Conversely, China has illustrated the benefits of the East Asian approach by decreasing rural women’s exposure to biomass fuel from 67% to 28% through the introduction of the PDE4 inhibitor crisaborole^[21]^ and the “clean stoves” rural energy transformation, while also raising the rate of grassroots pulmonary function testing to 67%.^[22]^ Global efforts should focus on high-burden regions such as South Asia, advocating for an integrated approach that comprises “environmental governance – grassroots screening – culturally tailored tobacco control” to mitigate regional disparities.

This research analyzes the dimensions of COPD burden – specifically ASIR, ASMR, and ASDR – across various SDI regions, breaking down the contributing factors of population aging, population growth, and changes in epidemiology. Globally, the primary influences behind the rise in COPD statistics are population growth and aging. While disease prevention and treatment strategies have contributed to reducing the burden of COPD, their effectiveness remains somewhat constrained. Notably, population aging and growth in low-middle- and middle-SDI regions exert a significant positive influence on the global COPD burden, likely attributable to outdated lifestyles and insufficient healthcare resources, which fail to adequately address the health demands of the elderly demographic. In a study conducted in Meru County, Kenya (2025), it was reported that 95% of individuals with COPD had not received an earlier diagnosis, and the prevalence of pulmonary function testing in primary healthcare facilities was under 10%.^[23]^ Similarly, the Vietnam National Aging Survey (2022) revealed that 20.2% of patients with chronic conditions, including COPD, suffered from depression. Rural communities encounter heightened risks due to inadequate healthcare resources (OR = 1.83).^[24]^ Additionally, population growth and aging in middle- and high-SDI regions have led to an increase in the ASMR associated with COPD; however, the establishment of effective prevention and management frameworks has considerably mitigated the epidemiological ramifications for various COPD metrics. For instance, after Turkey assigned COPD management to family physicians, there was an annual decline of 2.1% in ASMR. In 2020, Iran implemented a 30% increase in tobacco taxes, which resulted in a 46% decrease in the male smoking-related population attributable fraction, aligning with the global average.^[25]^

This research conducted an analysis of risk factors using data on DALYs and fatalities associated with COPD. The findings indicated that 3 predominant risk factors – smoking, environmental particulate matter, and household air pollution from solid fuels – together accounted for over 70% of the global burden of COPD in terms of both DALYs and fatalities. However, when stratifying regions by the SDI quintiles, significant variations in the data and distribution of DALYs and deaths were observed across different SDI levels. In regions classified as low SDI and low-middle SDI, household air pollution emerged as the primary risk factor for COPD DALYs and fatalities, accounting for as much as 55.0%. This trend suggests that a substantial portion of the population in these areas continues to rely on solid fuels for cooking and heating. The de Oca et al, through its GBD study, highlighted that coal use, post-tuberculosis health complications, and indoor air pollution (associated with biomass fuel exposure) in countries such as Kenya and Nigeria have contributed to a high incidence of COPD.^[22]^ To effectively alleviate the disease burden in this population, it is crucial to implement appropriate interventions that promote the adoption of clean energy and the renovation of stoves. The transition from traditional solid fuels to cleaner energy sources can reduce the likelihood of developing COPD by 22% (risk ratio = 0.78).^[26]^ Furthermore, chimney renovations have proven to be an effective strategy for mitigating coal-related COPD.^[27]^ By directly addressing household air pollution, the risk of exposure within the community can be significantly reduced, thereby improving COPD rates. In regions with a high- or high-middle SDI, smoking contributes to over 40% of COPD-related DALYs and fatalities, with smoking notably facilitating the onset and exacerbation of COPD. Even minimal smoking behavior (defined as <20 pack-years) is associated with 29% to 39% of new COPD cases and exhibits a synergistic effect in conjunction with airway hyperresponsiveness (interaction *P* < .001).^[28]^ Consequently, it is essential to continually enhance tobacco control laws, increase tobacco taxes, and vigorously promote smoking cessation services in these areas. Furthermore, while the average effect of extreme heat on global COPD DALYs and mortality is under 1%, this figure can rise to 1.4% and 1.1% in regions with a low SDI. As global warming continues to advance, it is anticipated that the frequency and severity of extreme heat incidents will increase, amplifying their effects on the COPD burden. For example, an analysis indicated that within a week after experiencing a heatwave, the risk of all-cause mortality among patients with COPD increased by 14% (incidence rate ratio = 1.14, 95% confidence interval: 1.09–1.20).^[29]^ Additionally, there is a compounded effect between elevated temperatures and PM_2_._5_; specifically, for each 10 μg/m^3^ increase in PM_2_._5_ levels, the likelihood of emergency department visits for COPD during periods of high heat increases by an additional 11%.^[30]^ Hence, it is crucial to implement public health initiatives that focus on extreme heat. To alleviate the potential detrimental effects of future climate change on COPD, strategies such as increasing urban green space by 30% can reduce the incidence of heat-related respiratory conditions by up to 23%.^[30]^ Moreover, integrating environmental considerations into treatment decisions has led to a 35% increase in the adoption of dry powder inhalers among COPD patients, alongside a notable decrease in the rates of acute exacerbations.^[31]^

This research revealed that the prevalence was approximately equal between the 2 sexes, although females displayed a slightly reduced prevalence compared to males. A positive association with age was observed for both ASMR and ASDR, and within similar age categories, males typically outnumbered females. As individuals aged, the gender gap became increasingly evident, with males significantly surpassing females in numbers. Furthermore, this investigation examined the variations in ASMR/ASPR, incidence rates, and fatalities from 1990 to 2021 across different genders. Over time, both incidence rates and mortality gradually rose, with males consistently outnumbering females. ASMR exhibited a declining trend, while ASPR remained relatively constant but showed signs of decreasing. Males showed greater disease severity and poorer prognostic outcomes, potentially linked to lower smoking exposure or better adherence to treatment among female patients.^[32]^ There were no reported cases of COPD among individuals aged 0 to 14 years, likely because many pediatric lung conditions are misidentified as more common illnesses such as bronchitis, pneumonia, or asthma.^[33]^ Nevertheless, the incidence of COPD in women younger than 45 years surpassed that in men, possibly owing to an increasing smoking rate among females and variations in biological vulnerability.^[34,35]^

The joinpoint regression model demonstrated a clear and steady decline in the ASMR for both men and women from 1990 to 2021, with particularly significant reductions noted among men. Notably, the most rapid decrease in ASMR for women occurred between 2004 and 2007, while for men, the steepest decline was observed from 2003 to 2006. This positive trend is likely linked to the implementation of effective tobacco control policies, advancements in medical standards, and the encouragement of healthier lifestyle choices. Such interventions have collectively contributed to the reduction in mortality rates over the years.^[36]^

The APC analysis further supports the observation of a sustained decline in incidence rates among birth cohorts that emerged after the 1960s. This trend implies that the tobacco control measures and workplace safety regulations enacted over the last 3 decades have resulted in significant cohort effects, demonstrating their lasting impact on public health.^[36]^ These findings underscore the importance of maintaining and enhancing such policies to continue reducing disease incidence. Additionally, the disease burden illustrates a clear dependency on age, with individuals aged over 40 experiencing the majority of health challenges. As age progresses, particularly among those over 80, the incidence of disease continues to rise. This pattern can be attributed to several factors, including diminishing physical capabilities, weakened immune responses, cumulative exposure to various health risks, and the presence of comorbidities commonly seen in the elderly population.^[37]^ Given this context, it is imperative to devise more specialized models that focus on pulmonary rehabilitation, the management of comorbid conditions, and care tailored to meet the needs of the elderly.^[38]^ By addressing these specific requirements, healthcare systems can improve outcomes and enhance the QoL of older adults facing these challenges.

This research employs the BAPC model to forecast that the global ASIR, ASMR, and ASDR of COPD will either stabilize or experience a slight decrease from 2021 to 2025. Zhu et al applied the BAPC model to anticipate a downward trajectory in ASIR extending from 2021 through 2050^[39]^; concurrently, Zhai et al utilized the same model to predict a decline in the ASMR and ASDR of CRD from 2021 to 2035.^[40]^ The findings from these investigations largely align with the predictions of the present study, thereby affirming the reliability of the BAPC model in capturing trends over both short-term (2021–2025) and long-term (up to 2035/2050) periods. Additionally, these findings reinforce the notion that the global burden of COPD has transitioned into a phase characterized by a “plateau-gradual decline,” with no immediate uptick expected in the short term. This provides substantial evidence to inform the development of prevention and control strategies for the years 2021 to 2025 across various nations.

The strengths of this study are primarily reflected in 5 aspects. First, the data foundation is robust and comprehensive in coverage. By leveraging the GBD 2021, this study systematically integrates the incidence, mortality, DALYs, and 8 major categories of risk factors for COPD across 204 countries and regions over a 32-year span, achieving, for the first time, a panoramic depiction of the dynamic burden of COPD on a global scale. Second, the methodologies employed are diverse and mutually validating. Decomposition analysis quantifies the independent contributions of population aging, population growth, and epidemiological transition; Joinpoint regression identifies trend inflection points; and the APC and BAPC models not only trace historical cohort effects but also project forward to 2050, significantly enhancing the robustness and foresight of the conclusions. Third, the stratified analysis of risk factors was conducted for the first time according to SDI quintiles, revealing a differentiated attribution pattern: “low SDI dominated by household solid fuels and high-middle SDI dominated by smoking,” which provides a quantitative basis for precise intervention. Fourth, this study is the first to discover and elaborate in detail on the “inverted V-shaped” relationship between the ASR of COPD and SDI, suggesting that reliance solely on economic growth cannot automatically lead to a reduction in disease burden; rather, it requires simultaneous advancement in environmental governance and medical system construction. Finally, the research results are directly oriented toward policy transformation: whether it is the cost-effectiveness estimation of promoting clean stoves in Africa or the implementation path for increasing the rate of primary pulmonary function screening in East Asia, both provide actionable and quantifiable guidelines.

Based on the aforementioned research, differentiated action plans are proposed for the 5 SDI regions. Low-SDI countries should prioritize the elimination of household solid-fuel pollution, which can be accelerated through a combination of international funding and local subsidies to promote the adoption of liquefied petroleum gas or solar cookers, supplemented by community health education. Low-middle-SDI regions need to simultaneously control environmental particulate matter and occupational exposure; for instance, by implementing total industrial emission controls in South Asian cities, mandating the use of dust masks in the textile and mining industries, and establishing an annual lung function monitoring system for workers exposed to dust. Middle-SDI countries should place equal emphasis on tobacco control and grassroots screening: on one hand, by significantly increasing tobacco taxes and graphic health warnings to reduce smoking rates; on the other hand, by equipping township health centers with portable spirometers and training grassroots doctors in simple COPD screening procedures. High-middle-SDI regions face the challenge of rapid aging and are advised to include COPD in the family doctor contracted service package, promote the “pulmonary rehabilitation + chronic disease co-management” model, and leverage medical insurance payment reforms to incentivize early intervention at the grassroots level. High-SDI countries need to focus on emerging risks such as ozone pollution and extreme heat events, which can be mitigated through urban greening, high-temperature health warning systems, and the promotion of low environmental-impact dry powder inhalers, thereby further reducing the risk of acute exacerbations in sensitive populations.

## 5. Limitations

This study acknowledges several limitations that could influence its findings. First, while the research utilized a multidimensional analysis based on the GBD 2021 data, the overall accuracy and completeness of these data depend on the quality of reporting from individual countries. It is important to recognize that some low- and middle-income countries may encounter challenges such as underreporting or inadequate diagnostic practices. In these regions, spirometry is often inaccessible in primary care settings, and COPD may be frequently misclassified as chronic bronchitis, asthma, or other respiratory conditions, leading to a systematic underestimation of the true disease burden. This issue is particularly concerning because the observed disparities across SDI strata may therefore be conservative; the actual gaps in COPD burden between high-SDI and low-SDI regions could be even larger than reported here. These challenges underscore the urgent need for improved data collection, standardized diagnostic protocols, and enhanced reporting systems in these regions. Second, the BAPC model employed in this study is designed to offer long-term predictive capabilities. However, the projections made for the next 3 decades are constrained by the stability of the model’s assumptions. The model’s framework inherently assumes that historical trend patterns will persist into the future and does not fully accommodate potential disruptions caused by nonlinear factors, which may include sudden public health emergencies (such as the COVID-19 pandemic), policy shifts (e.g., radical tobacco taxation or clean energy mandates), or extreme climate incidents. These unforeseen elements could significantly alter the trajectory of COPD burden in ways that the model cannot predict. Therefore, our projections should be interpreted as scenario-based forecasts under current trend assumptions rather than definitive predictions, highlighting the necessity for more adaptable modeling approaches that can account for such unpredictable variables. Third, in the analysis of risk factor attribution, the study integrated 8 primary risk factors. However, it is noteworthy that certain emerging risks – such as exposure to microplastics and imbalances in the lung microbiome resulting from antibiotic misuse – were not considered in this research. The exclusion of these risks may lead to an underestimation of their actual impact on COPD, suggesting that future studies should expand the range of risk factors analyzed to provide a more comprehensive understanding of the disease’s etiology. Fourth, this study faced limitations due to the absence of detailed individual-level data for certain countries within the GBD database. Consequently, it was unable to conduct a comprehensive exploration of micro-level factors, such as disparities between urban and rural areas, variations in occupational exposure, or genetic predispositions that may influence the burden of COPD. The lack of this nuanced analysis restricts the study’s capacity to provide tailored insights that could address these specific contributing factors. Finally, it is crucial to note that this study did not include a separate examination of acute exacerbation events related to COPD. Given that acute exacerbations are recognized as pivotal drivers of disease progression, hospitalization, mortality, and substantial healthcare expenditures, their omission from our analytical framework may limit the direct translational value of our findings for clinical management and resource allocation. We acknowledge this as a significant gap and plan to prioritize the incorporation of exacerbation-related outcomes in our future research to provide more clinically actionable evidence.

## 6. Conclusion

According to the findings from the GBD 2021 study, the global age-standardized incidence, mortality, and DALY rates for COPD have consistently declined from 1990 to 2021. However, the total numbers of cases, fatalities, and DALYs associated with COPD are still on the rise due to population growth and an increasingly aging demographic, indicating a shift in the disease burden from “high mortality” to “high prevalence.” An inverted “V” shaped correlation exists between the SDI and disease burden: within an SDI range of 0.4 to 0.6, economic progress paradoxically correlates with an increased burden; conversely, when the SDI exceeds 0.7, there is a gradual alleviation of the burden linked to improvements in environmental management and healthcare conditions. Globally, factors such as smoking, exposure to ambient particulate matter, and household air pollution account for over 70% of deaths and DALYs, with low-SDI regions primarily affected by household pollution (55%), while high-SDI areas are significantly impacted by smoking (over 40%). The increase in disease burden is particularly pronounced in South Asia and sub-Saharan Africa. Projections utilizing the BAPC model indicate that from 2022 to 2050, global ASRs for incidence, mortality, and DALYs are likely to stabilize or experience slight reductions. Nevertheless, the overall burden in low-to-middle-SDI nations is expected to continue rising due to population growth and aging effects. To achieve the United Nations’ target for 2030 regarding the reduction of premature deaths from noncommunicable diseases, it is essential to implement a comprehensive intervention strategy in low- and middle-SDI countries, incorporating a tripartite approach that includes “clean energy, community screenings, and culturally relevant tobacco control.” Implementing targeted strategies for prevention and control that effectively integrate tobacco regulation, the advancement of clean energy, and equitable access to primary healthcare is crucial for managing the future disease burden associated with COPD.

## Acknowledgments

We express our gratitude to the Institute for Health Metrics and Evaluation staff and its collaborators who prepared these publicly available data. The authors acknowledge all of the persons involved in the GBD Collaborator Network: Junhao Ma and Luna Zhao.

## Author contributions

**Conceptualization:** Wanting Wang.

**Data curation:** Junhao Ma, Xinxin Zhang, Kaiqi Zhang.

**Funding acquisition:** Dong Liu, Luna Zhao, Chao Wu.

**Investigation:** Luyi Fang, Chao Wu.

**Methodology:** Hongding Zhao, Hanwen Zhang, Lang Wang, Wanting Wang, Man Luo, Yun Jia, Jinyang Li.

**Project administration:** Lin Chen, Hongding Zhao, Yun Jia.

**Resources:** Dong Liu, Luna Zhao, Lin Chen, Hongding Zhao, Jingkun Li, Yue Zhou.

**Software:** Yihao Zhang, Hanwen Zhang.

**Supervision:** Luna Zhao, Yihao Zhang, Junwei Wang, Kexin Cui, Yi Sun, Fangyi Zhao, Sijie Huang.

**Validation:** Guizhen Lv, Junwei Wang, Yimiao Qiu, Ye Lui, Fangyi Zhao, Yuye He, Rui Zhao.

**Visualization:** Guizhen Lv, Junhao Ma, Junwei Wang, Yuye He, Hongzheng Liu.

**Writing – original draft:** Dong Liu, Luna Zhao.

**Writing – review & editing:** Dong Liu, Luna Zhao.
